# Frozen Shoulder as a Metabolic Signal: Advocating Routine HbA1c Screening in Atraumatic Cases

**DOI:** 10.7759/cureus.86999

**Published:** 2025-06-29

**Authors:** Raktim Swarnakar

**Affiliations:** 1 Physical Medicine and Rehabilitation, National Cancer Institute, Jhajjar Campus, All India Institute of Medical Sciences, New Delhi, IND

**Keywords:** diabetes type 2, glycated hemoglobin (hba1c), idiopathic adhesive capsulitis, idiopathic frozen shoulder, musculoskeletal shoulder pain

## Abstract

Frozen shoulder, also known as adhesive capsulitis, is often regarded as a localized musculoskeletal disorder. However, it may also be an early indicator of underlying metabolic dysfunction, particularly diabetes or prediabetes. Many patients presenting with atraumatic, progressive shoulder stiffness are found to have elevated HbA1c levels despite normal fasting and postprandial glucose values. Diabetes-related tissue changes, such as collagen glycation and microvascular compromise, may contribute to the development of capsular fibrosis. Recognizing this, routine HbA1c screening in such presentations can aid early diagnosis of dysglycemia and enable timely intervention. This approach may promote interdisciplinary care and improve both systemic and shoulder outcomes. Clinicians should consider frozen shoulder within a broader metabolic-inflammatory framework, especially in middle-aged patients with persistent symptoms and no clear alternative diagnosis.

## Editorial

As clinicians, red flags, physical signs, and patterns of symptoms often guide diagnostic and therapeutic strategies. Yet, the body often signals systemic dysfunction through subtler signs that may initially appear unrelated. One such subtle yet significant clinical presentation is atraumatic, insidious-onset shoulder stiffness, commonly diagnosed as adhesive capsulitis, also known as "frozen shoulder," which may serve as an early clinical clue to previously undiagnosed metabolic dysfunction.

A noticeable clinical trend has emerged wherein more than one-third of patients presenting with such shoulder dysfunction are found to have elevated HbA1c (hemoglobin A1c or glycated hemoglobin) levels, despite lacking a prior diagnosis of diabetes mellitus or any classical signs or symptoms such as polyuria, polydipsia, or unexplained weight loss [1]. In these individuals, HbA1c values frequently fall within the prediabetic (5.7%-6.4%) or diabetic range (≥6.5%), pointing toward an underlying dysglycemic state that had previously gone unrecognized [1].

Although the association between diabetes mellitus and adhesive capsulitis is well-documented in the literature, the clinical potential of shoulder dysfunction as a sentinel symptom of occult diabetes remains relatively less utilized in daily practice [2]. This highlights the need for a paradigm shift in how frozen shoulder is perceived, not merely as a local musculoskeletal issue, but as a possible manifestation of systemic metabolic imbalance.

Pathophysiologically, diabetes exerts deleterious effects on connective tissues through mechanisms such as non-enzymatic glycation of collagen (a process where excess glucose attaches to collagen without the aid of enzymes, which occurs more rapidly in diabetes and leads to the accumulation of substances called advanced glycation end-products), chronic low-grade systemic inflammation, and microvascular ischemia. These changes lead to structural remodeling, resulting in capsular fibrosis and reduced joint mobility, particularly in the glenohumeral joint. Given the shoulder's complex anatomy and biomechanical demands, it becomes a vulnerable target for such metabolic insults. Among metabolic biomarkers, HbA1c and serum cholesterol levels (total cholesterol and low-density lipoproteins) showed a very strong association with frozen shoulder, suggesting a broader interplay between musculoskeletal and metabolic health [3].

Moreover, emerging evidence supports the view that frozen shoulder fits within a broader metabolic-inflammatory framework (characterized by chronic low-grade inflammation and fibrotic inflammatory processes induced by systemic metabolic dysfunction), rather than merely representing a localized orthopedic condition [3]. This perspective aligns with evolving evidence that systemic inflammation, insulin resistance, and dyslipidemia may collectively contribute to the development and persistence of shoulder joint fibrosis. Importantly, individuals with diabetes not only face a higher risk of developing adhesive capsulitis but also experience worse functional outcomes, delayed recovery, and a higher likelihood of recurrence when compared to non-diabetic counterparts [4].

**Figure 1:**
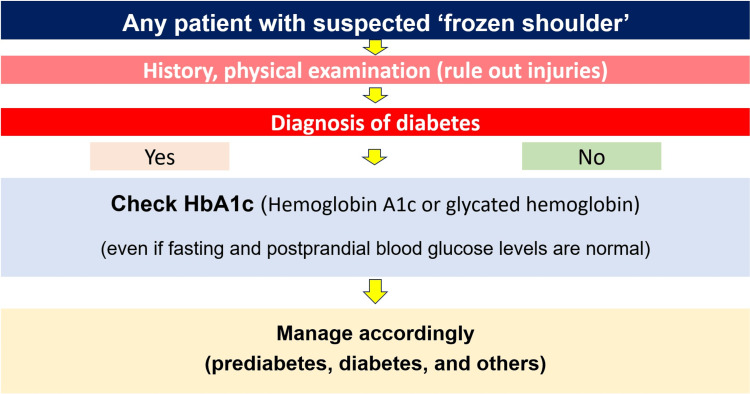
Schematic diagram illustrating the incorporation of HbA1c screening in the approach to suspected frozen shoulder.

Given the emerging evidence, routine HbA1c screening may be worth exploring in patients presenting with non-traumatic, progressively worsening shoulder stiffness, particularly among middle-aged and older adults, though further studies are needed to determine whether this approach is clinically prudent and cost-effective (Figure 1).

Early detection of dysglycemia facilitates timely lifestyle and pharmacological interventions that may alter the course of both the systemic condition and the shoulder pathology. Identifying and treating the metabolic disorder in its early stages could improve both systemic health and local tissue recovery, reducing chronicity and long-term disability.

Such a clinical approach also promotes interdisciplinary collaboration. Rehabilitation specialists, endocrinologists, and primary care physicians can work in tandem to develop integrated management plans addressing both the mechanical limitations and metabolic dysregulation.

It is important to acknowledge that not all cases of shoulder dysfunction originate from metabolic causes. Differential diagnoses, such as rotator cuff pathology, cervical radiculopathy, postural syndromes, or inflammatory arthropathies, must be carefully ruled out through clinical assessment and, where appropriate, imaging. However, in patients with an atraumatic, stiffness-predominant presentation and no obvious local pathology, screening for HbA1c becomes a valuable, low-risk, and informative tool that may substantially influence clinical decision-making [5].

Musculoskeletal complaints, especially those that appear without a clear external cause, can sometimes serve as early indicators of systemic disease. The shoulder, in particular, due to its complex biomechanics and high functional demand, may manifest early signs of metabolic compromise. Recognizing this possibility and incorporating routine HbA1c screening in select patients, such as middle-aged adults and those presenting with atraumatic shoulder stiffness, can significantly improve early detection of prediabetes or diabetes, thereby enabling timely intervention and holistic care.

Frozen shoulder may not always be an isolated musculoskeletal issue. In many cases, it may be the first signal of underlying metabolic dysregulation, particularly diabetes or prediabetes. Incorporating HbA1c screening into the diagnostic workup for non-traumatic, insidious shoulder stiffness is a simple yet impactful clinical practice that may aid in early diagnosis, guide comprehensive management, and improve long-term outcomes; however, caution must be exercised to avoid unnecessary testing, and further research is warranted to substantiate these observations with larger datasets. As healthcare moves toward more proactive and integrated models of care, attention to such subtle clinical cues can play a vital role in bridging musculoskeletal and metabolic health.
